# Global research trends in sarcopenia: a bibliometric analysis of exercise and nutrition (2005–2025)

**DOI:** 10.3389/fnut.2025.1579572

**Published:** 2025-05-16

**Authors:** Runqian Zhang, Jiaxin Wang, Huijuan Xi, Yue Cheng, Bei Han

**Affiliations:** ^1^Health Science Center, Xi’an Jiaotong University, Xi’an, China; ^2^School of Public Health, Health Science Center, Xi’an Jiaotong University, Xi’an, China

**Keywords:** sarcopenia, exercise, nutrition, bibliometrics, visualization

## Abstract

**Objectives:**

This study aimed to evaluate the current research landscape, identify emerging areas of interest, and provide scientific insights for further research in exercise and nutrition for sarcopenia.

**Methods:**

A comprehensive bibliometric analysis was conducted using publications retrieved from the Web of Science Core Collection and SCOPUS between January 1, 2005 and January 1, 2025, focusing on exercise and nutritional interventions for sarcopenia. CiteSpace and VOSviewer were employed to visualize research trends through analysis of annual publications, keyword evolution, journal contributions, author networks, country/regional distributions, institutional collaborations, citation patterns, and high-frequency terminology.

**Results:**

The analysis included 886 publications demonstrating a consistent upward trajectory in annual output. Geographical shifts revealed a transition of research leadership from traditional centers in the United States and Europe to emerging Asian contributors, particularly China. High-frequency keywords analysis identified core concepts including “Skeletal Muscle” (Betweenness Centrality Degree, BCD 0.13), “Resistance Exercise” (BCD 0.11), and “Muscle Strength” (BCD 0.13), with nutritional components “Dietary Protein” (BCD 0.19), “Vitamin D” (BCD 0.14), and “Amino Acids” (BCD 0.18) forming distinct research clusters. Cluster analysis revealed five thematic domains: Protein Metabolism (Cluster 1), Body Composition Assessment (Cluster 2), Resistance Training Modalities (Cluster 3), Frailty Syndromes (Cluster 4), and Metabolic Regulation (Cluster 5). Temporal keyword evolution showed a paradigm shift from foundational terms (“human skeletal muscle”, “amino acids”) to clinical outcome measures (“gait speed,” “physical function,” “inflammation”) and mechanisms.

**Conclusion:**

The research trend in sarcopenia is currently shifting from symptoms to underlying mechanisms. Meanwhile, the focus of exercise and nutritional interventions for sarcopenia is moving toward addressing the disease burden and health management of multiple chronic diseases associated with sarcopenia.

## 1 Introduction

Sarcopenia, a progressive and generalized skeletal muscle disorder involving the accelerated loss of skeletal muscle mass and function (1), is observed in many species, including humans. In the past 20 years, many international groups have contributed to the development of conceptual and operational definitions of sarcopenia, which including European Society for Clinical Nutrition and Metabolism (ESPEN) (2), European Working Group on Sarcopenia in Older People (EWGSOP) (3), International Working Group on Sarcopenia (IWGS), Asian Working Group for Sarcopenia (AWGS) (4), Sarcopenia Definitions and Outcomes Consortium (SDOC) (5), Global Leaders Initiative in Sarcopenis (GLIS) (6), and et al. Now the conceptual definition of sarcopenia encompasses muscle mass, strength and muscle-specific strength as its ‘components’, and impaired physical performance has been recognized as an ‘outcome’ rather than a ‘component’ of sarcopenia.

As a chronic muscle degenerative disease, sarcopenia is a significant causes and manifestations of the gradual decline of physiological functions in the elderly (7). Sarcopenia has been associated with metabolic impairment, obesity, physical disability and malnutrition (8). The aetiology of sarcopenia is complex and poorly understood, with the underlying molecular mechanisms remaining largely unelucidated (9). Recent evidences indicate a potential link between chronic low-grade inflammation and sarcopenia, a condition characterized by the loss of muscle mass, strength, and function (10).

This disease is closely associated with the process of aging and is classified as an age-related disease (11). It is estimated that sarcopenia affects approximately 30% of individuals aged 65 and over, with a prevalence ranging from 50 to 60% among those aged 80 and above (12). As sarcopenia progresses, patients may experience muscle weakness, falls, fractures, and other complications, which have a severe impact on their quality of life and potentially reduce life expectancy, increasing burden of disease (13). The global elderly population is projected to reach two billion by 2050, in line with overall population growth. Furthermore, it is anticipated the prevalence of sarcopenia will increase, resulting in a concomitant rise in associated health issues (14). Therefore, there is an urgent need to develop and optimize clinical strategies for sarcopenia (15). Current clinical strategies for sarcopenia are primarily based on exercise and nutritional interventions. The most common employed training modalities encompass resistance exercise, aerobic exercise, a combination of both, balance training, flexibility exercises, and additional complementary strategies. These training modalities positively influence muscle mass and strength in patients with sarcopenia (16). Nutritional supplements including whey protein, branched chain amino acids, vitamins (Vitamin D, Vitamin C, B-Vitamins), minerals (calcium, selenium, magnesium), omega 3 fatty acids (EHA, EPA), and dietary regimes, like Mediterranean diet, were specifically designed to improve muscle health, and proved to prevent age-related deterioration in strength and function (17). Exercise and nutritional interventions are effective in preventing sarcopenia and facilitating rehabilitation. Evidence from several studies indicates that integrating exercise with nutritional interventions is a more effective approach (18–20). It is therefore essential to provide a comprehensive overview of the research on exercise and nutrition for the treatment of sarcopenia.

The bibliometric analysis and visualization tools of CiteSpace and VOSviewer were used to analyze publications and provide researchers with information on the evolution of research directions and the frontiers of development in a specific target area of study (21, 22), followed the preliminary guideline for reporting bibliometric reviews of the biomedical literature (23). However, there is a lack of bibliometric analysis on research of exercise, nutrition for sarcopenia. The aim of this review was to rapidly and accurately identify the research hotspots and the global research trajectory, thereby providing essential references for researchers in this field to gain insight into the cutting edge and focal points of sarcopenia.

## 2 Materials and methods

### 2.1 Data sources and search strategies

**Figure 1:**
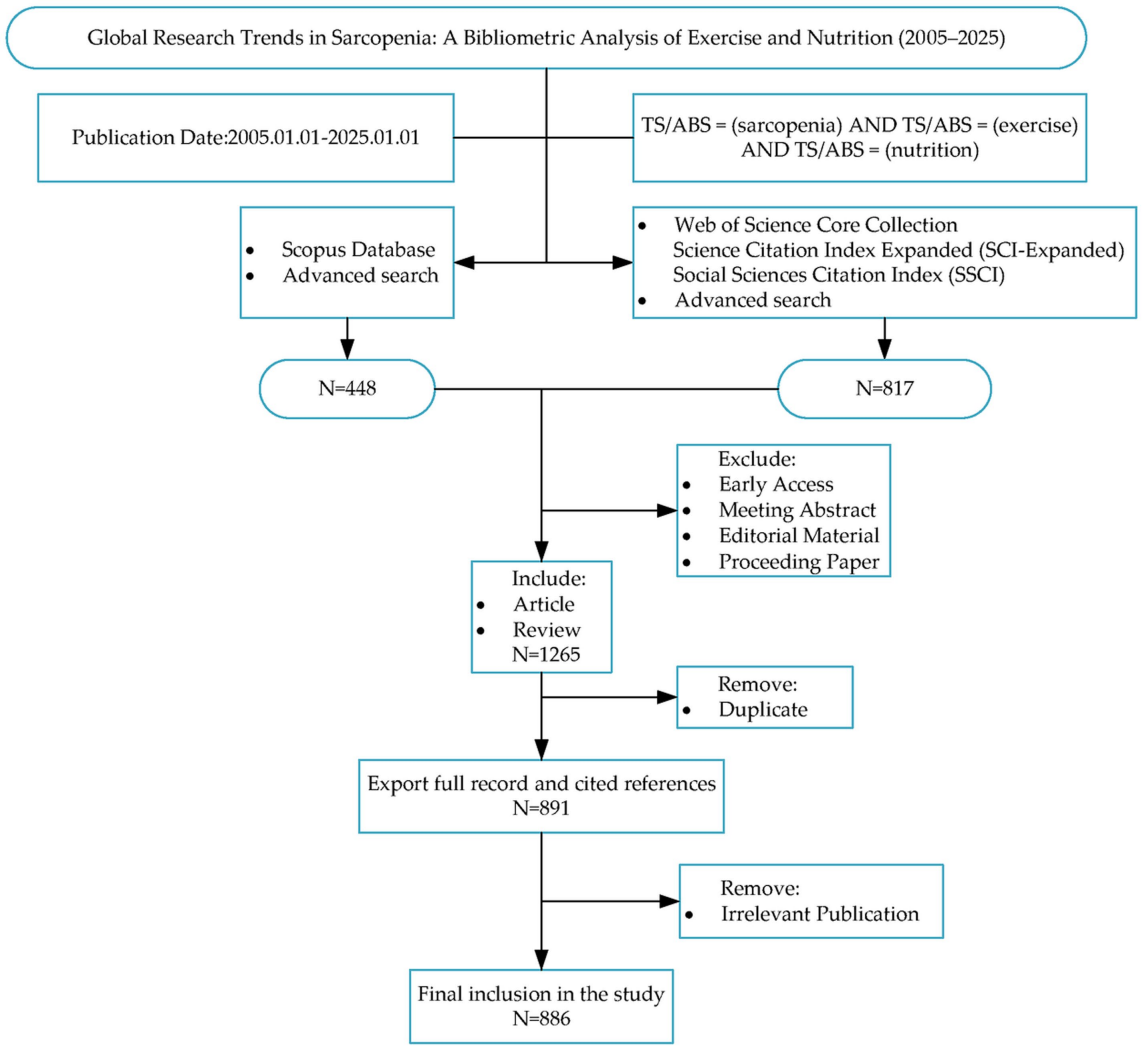
Flow chart illustrating the scientometric analysis process.

The Web of Science Core Collection (WOSCC) and SCOPUS were utilized as data source. All publications were retrieved from the Science Citation Index Expanded (SCI-E) and Social Sciences Citation Index (SSCI) on January 14, 2025, to avoid bias caused by database updates. The search strategy included the following search terms: TS/ABS = (sarcopenia) AND TS/ABS = (exercise) AND TS/ABS = (nutrition); The publication type was limited to “Article” and “Review” to ensure the representativeness of the included studies, with “English” as the designated language; The temporal scope for data retrieval was set between January 1, 2005 and January 1, 2025 in order to more accurately analyze the current status, hotspots and frontiers trends of sarcopenia, focused on exercise and nutrition, yielding a total of 886 publications. Subsequently, the relevant publications were carefully selected, and the “Full Record and Cited References” were exported in a plain text file format, including keywords, author information, citation data, and other relevant details. Figure 1 elucidates the process of scientometric analysis process and provides additional details.

### 2.2 Bibliometric and statistical analysis

The screened literature was standardized, statistically analyzed, and visualized using CiteSpace 6.1.R, VOSviewer 1.6.20, and Sigmaplot 10.0. Both VOSviewer and CiteSpace can generate maps that shows clusters of related terms. The size of each term on the map indicates its importance, and the thickness of the lines between the terms represents the strength of relationship between terms. This methodological approach was designed to construct a comprehensive visual and analytical atlas of the domain focusing on exercise and nutritional interventions for sarcopenia, complemented by pertinent statistical data. Curve Estimation in SPSS 25.0, a least squares-based regression analysis that supports 11 different curve models (e.g., linear, composite, and logistic regression models), was used to predict future data points or analyze trends in time series data. The curve estimation function is particularly suitable for time series data, especially when trends (e.g., growth, decay, or cyclical changes) were observed over time.

The clustering algorithm used by VOSviewer is a network clustering method similar to Modularity. The specific principle is as follows:


$$V(c_{1},\cdots ,c_{n})=\frac{1}{2m}\sum_{i<j}\delta (c_{i},c_{j})w_{\mathrm{ij}}(c_{\mathrm{ij}}-\gamma \frac{c_{i}c_{j}}{2m})$$

In the formula, $w_{\mathrm{ij}}=\frac{2m}{c_{i}c_{j}}$ , $c_{i}$ represents the cluster to which element i belongs, and the equation value represented by $\delta (c_{i},c_{j})$ is 1 (if $c_{i}=c_{j}$ ) or 0, γ represents the clustering resolution, $\gamma =\{\begin{array}{c} 0,x_{i}=x_{j}\\ \frac{1}{d_{\mathrm{ij}}},x_{i}\neq x_{j} \end{array}$ , When $w_{\mathrm{ij}}$ and γ in the above formula are set to 1, the network clustering algorithm is consistent with modularity. We conducted keyword clustering analysis using the following parameters, set the minimum occurrence frequency of each term to be included in the analysis to the smallest value that ensures the number of nodes in the generated graph is less than or equal to 60, use the VOSviewer default settings for the remaining parameters (22, 24, 25).

During analysis, Kleinberg’s burst detection algorithm in CiteSpace was used to identify emerging research frontiers through keyword burst analysis (21, 22, 26). The keyword burst detection feature highlights emerging keywords as indicators of research hotspots and trends across different periods. Betweenness centrality, which quantifies the number of times a node (or edge) acts as a bridge along the shortest path between other nodes, was used to measure a node’s influence within the network. The degree of influence exerted by a node within the visual network is quantified by its betweenness centrality degree (BCD), higher BCD greater influence. And nodes with BCD exceeding 0.1 are considered as pivotal or transformative within a given field. In the visualized network graphs, size of nodes is proportional to the number of publications or the frequency of keyword occurrence. Node size also reflects national/regional publication counts, and the thickness of the connection indicates the depth of the cooperation between countries/regions. The colors indicate the average year of publications in the visualization of country/regional publications over time.

## 3 Results

### 3.1 Analysis of annual publications

**Figure 2:**
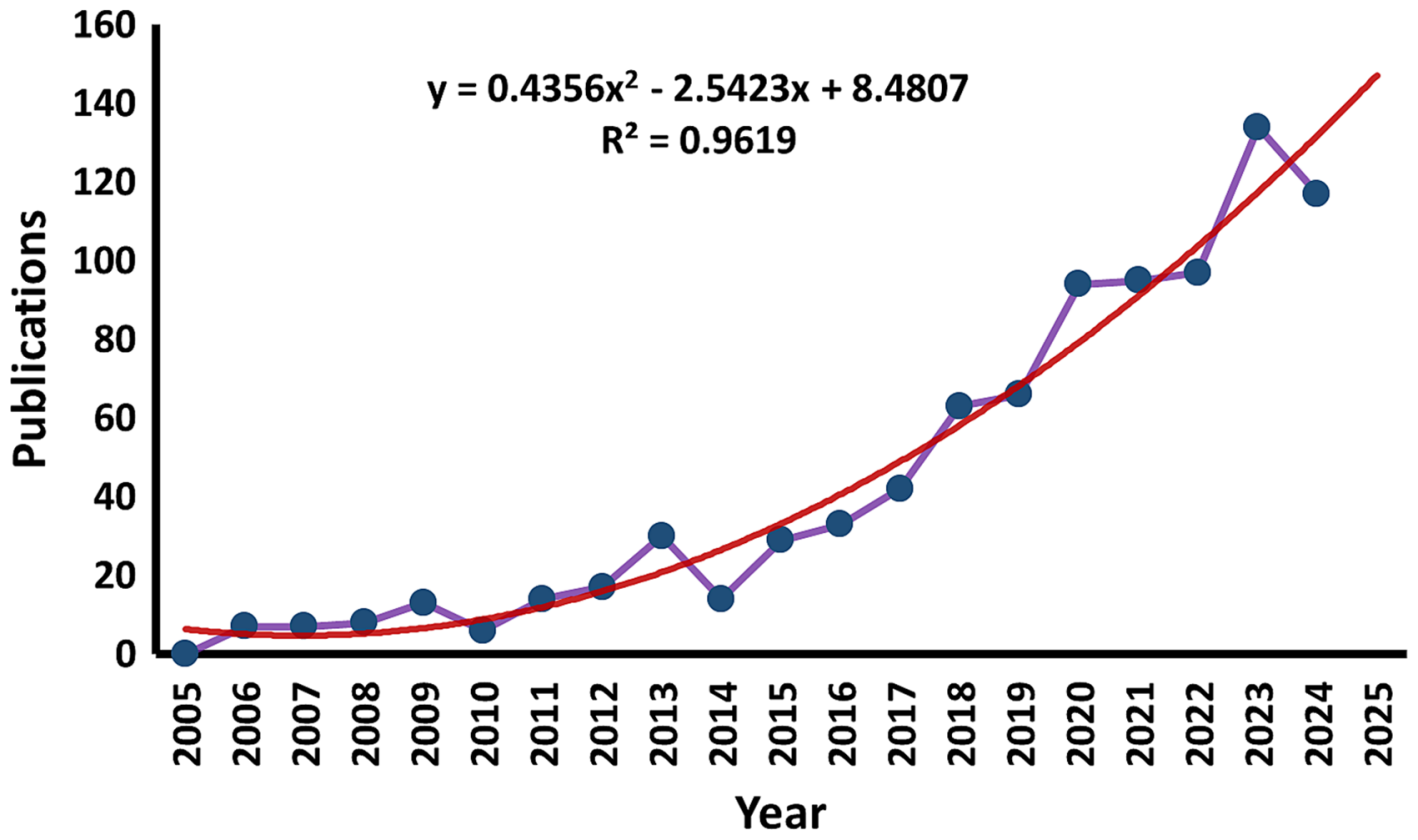
Annual publications and predicted curve from 2005 to 2025. The purple line is the observed data, and the red line is the predictive modeling generated data.

In the past 10 years, there were a total number of 886 publications focused on “sarcopenia, exercise, nutrition,” which including 601 articles (68%) and 285 reviews (32%). Figure 2 shows that there has been an upward trend in the number of publications over the last two decades. In this area publications in the past 20 years can be divided into 2 stages. The initial stage (2005–2014) was a steady period. The average number of publications was 12 per year, with the lowest number of publications being 0 publications in 2005 and the highest number being 30 publications in 2013. Although the number of papers varied at this stage, the overall trend was one of consistent growth. The second stage (2015–2024) was a sustained growth period. The average number of publications annually was 77 publications. The number of publications reached 134 in 2023. It was predicted that research achievements in nutrition and exercise for sarcopenia will continue to increase. By 2025, the expected annual publications on this field will reach approximately 132 ± 8.

### 3.2 Country/regional and institutional analysis

An analysis of the top 10 countries/regions in terms of number of publications and the top 10 countries/regions in terms of BCD was shown in Supplementary Table S1. The countries that have published the high number of publications in this field over the past decade are the USA (*n* = 148), South Korea (*n* = 101) and Japan (*n* = 99). At the same time, the distribution of countries in this field was uneven, and the top effect was pronounced. Scholars from several countries wrote most papers. Countries with high BCD are Germany (BCD = 0.73), Poland (BCD = 0.42) and Scotland (BCD = 0.38). Consolidation of the number of publications and BCD, countries with a strong presence in this field are the USA, South Korea, Germany, Sweden and Spain.

In Supplementary Table S2, the top five organizations are University of Texas System, the USA (*n* = 17), Catholic University of the Sacred Heart, Italy (*n* = 16), McMaster University, Canada (*n* = 16), IRCCS Policlinico Gemelli, Italy (*n* = 15) and University of Melbourne, Australia (*n* = 14). Among the top 10 institutions in terms of publications, 80% are comprehensive research universities. The organizations with highest BCD are University of Alberta, Canada (BCD = 0.114). Among the top 10 institutions in terms of centrality, the most numerous institutions were from Canada, with a share of 30%, and the share of comprehensive universities was 70%. Analysis in conjunction with visualization of inter-institutional relationships, suggested that the institutions with an influential position in this field are the University of Texas System (USA), the Uppsala University (Sweden), Karolinska Institutet (Sweden), CIBER - Centro de Investigacion Biomedica en Red (Spain) and University of Alberta (Canada).

**Figure 3:**
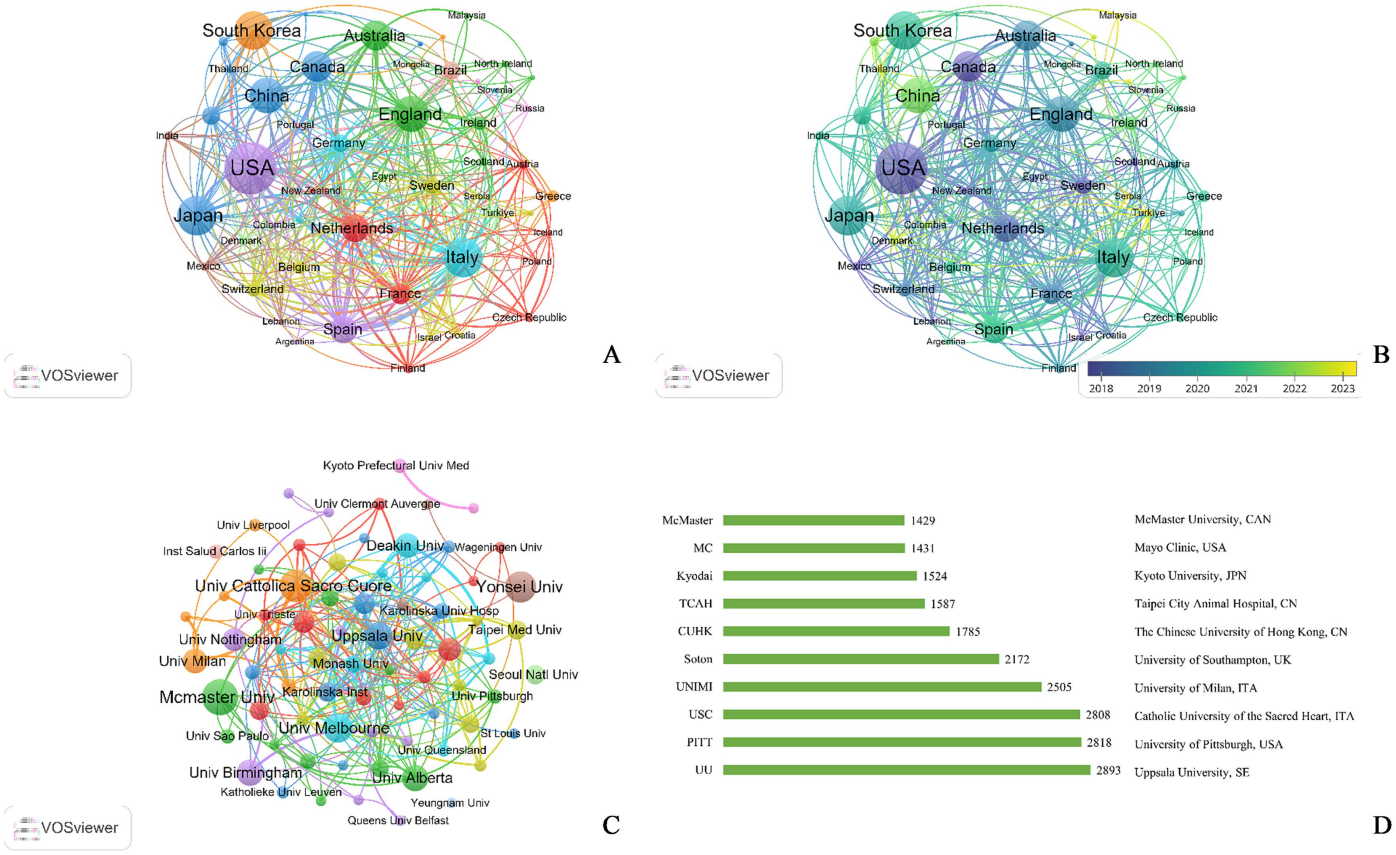
Network visualization of publications in exercise and nutrition for sarcopenia from 2005–2025. **(A)** Cooperation network of countries/regions; **(B)** countries with publication year, the colors of nodes and links indicates the average appearing year; **(C)** cooperation network of research institutions; **(D)** top 10 research institution citations.

A visual representation of the country/region contributions to the publications in this field was shown in Figure 3A, with 58 nodes representing 58 countries/regions that have conducted research in this field in the last two decades. In Figure 3B, the research hotspot in this field has shifted from the US and Europe countries to Asia, especially China, over time in the last two decades. The number of institutional publications reveals that 1,341 institutions have published at least one piece of research publication in this field. Only those institutions with a significant output, defined as having at least five publications, are displayed in Figure 3C. The size of the nodes corresponds to the document counts; the lines suggest the connections between institutes, and the thickness of the lines indicates the strength of the connection between institutes. In Figure 3D, institutions from Italy had the highest number among the top 10 bars in the number of citations to their institutions.

### 3.3 Journal analysis

Researchers can accurately understand the core journals in a topic by analyzing its source journals, which also serves as a reliable resource for further field research. The 886 publications were published in 378 journals in the last 20 years. There were 34 journals that had published more than five publications at least. The journal “Nutrients” (H-index 75) had published 76 publication, followed by “Clinical Nutrition” (H-index 121), 44 publication (Supplementary Table S3). The top 10 journals accounted for 27.65% of the total publications. Of the top 10 journals, all journals’ IF more than 3.0. With a maximum of 9.4, 2 journals had an IF >6.0. This shows that high IF journals are open to publishing exercise and nutrition for sarcopenia research.

**Figure 4:**
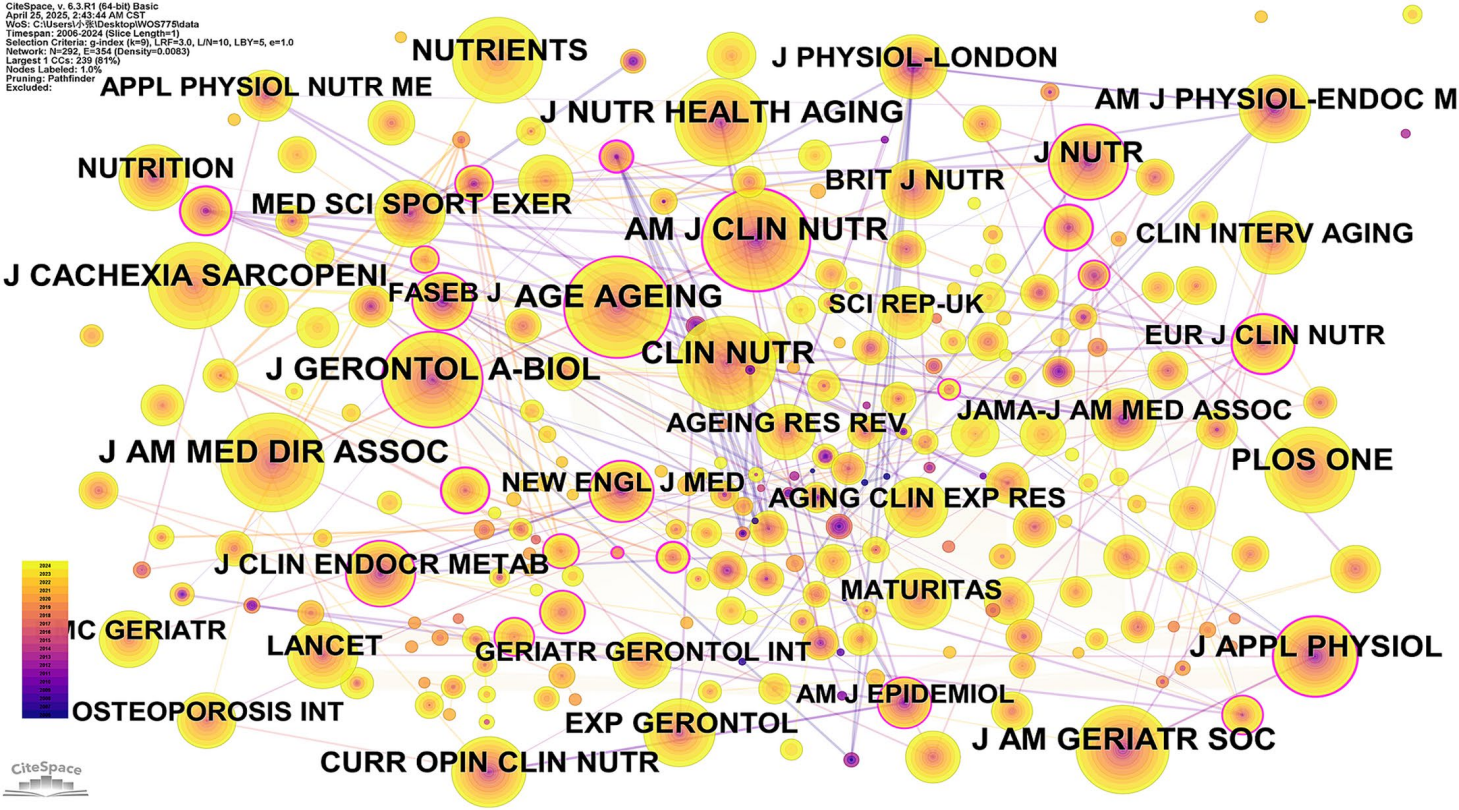
Journal citations visualization in research of exercise and nutrition for sarcopenia from 2005–2025. The size of the nodes represents the number of citations.

A citation map of the journals was generated, yielding 292 nodes and 354 links, and there had a total of 36 journals were cited more than 150 times (Figure 4). The most cited journal was “American Journal of Clinical Nutrition” (H-index 307) with 534 citations, followed by “Age and Aging” (H-index 124) and “Journals of Gerontology Series A – Biological Sciences and Medical Sciences” (H-index 168) with citations of 524 and 512, separately (Supplementary Table S4).

### 3.4 Analysis of cited publications

**Figure 5:**
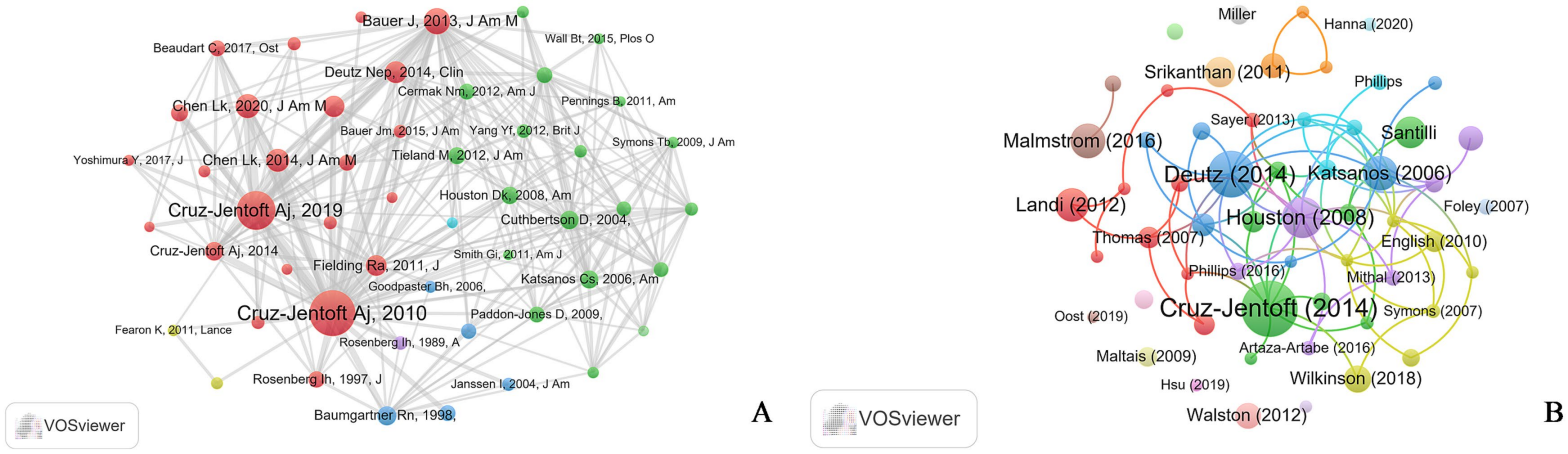
Network visualization in research of exercise and nutrition for sarcopenia from 2005–2025. **(A)** Network map of co-cited references, the size of the nodes represents the number of citations; **(B)** network map of global citations of documents, the size of the nodes represents the number of citations.

The number of citations is a frequently employed indicator of the impact of a given publication. High cited references lay the foundation and accelerate the development of research in the field. Publications with a high citation are considered to be authoritative within their research fields. Co-cited references were used to study the internal connections between literatures and depict scientific development’s dynamic structure. A visualization map of the references was generated using the VOSviewer software (Figure 5A). Among the 37,672 references, the most frequently cited reference is “Sarcopenia: European consensus on definition and diagnosis: Report of the European Working Group on Sarcopenia in Older People” by Cruz-Jentoft AJ, published in 2010 in “Age and Ageing,” and this publication has been cited 262 times in 770 publications (3).

The publication citation visualization map is shown in Figure 5B, and the top 10 most cited publications are shown in Table 1. Of the top 10 references, 6 references were articles and 4 were reviews. Of all 886 publications, 57 were cited more than 150 times, and the most frequently cited publication is “Prevalence of and interventions for sarcopenia in aging adults: a systematic review. Report of the International Sarcopenia Initiative (EWGSOP and IWGS),” with 1,380 citations, followed by “Protein intake and exercise for optimal muscle function with aging: Recommendations from the ESPEN Expert Group” (1,058 citations) and “Dietary protein intake is associated with lean mass change in older, community-dwelling adults: The Health, Aging, and Body Composition (Health ABC) Study” (882 citations).

**Table 1:** The top 10 cited publications of exercise and nutrition for sarcopenia from 2005–2025.

Rank	Publication title	Journal (H-index)	Publication type	Authors	Years	Number of citations
1	Prevalence of and interventions for sarcopenia in aging adults: a systematic review. Report of the International Sarcopenia Initiative (EWGSOP and IWGS)	Age and Aging (H-index 124)	Review	Cruz-Jentoft AJ et al	2014	1,380
2	Protein intake and exercise for optimal muscle function with aging: recommendations from the ESPEN Expert Group	Clinical Nutrition (H-index 121)	Article	Deutz NE et al	2014	1,058
3	Dietary protein intake is associated with lean mass change in older, community-dwelling adults: the Health, Aging, and Body Composition (Health ABC) Study	American Journal of Clinical Nutrition (H-index 307)	Article	Houston DK et al	2008	882
4	SARC-F: a symptom score to predict persons with sarcopenia at risk for poor functional outcomes	Journal of Cachexia Sarcopenia and Muscle (H-index 48)	Article	Malmstrom TK et al	2016	697
5	A high proportion of leucine is required for optimal stimulation of the rate of muscle protein synthesis by essential amino acids in the elderly	American Journal of Physiology Endocrinology and Metabolism (H-index 182)	Article	Katsanos CS et al	2006	694
6	Sarcopenia as a risk factor for falls in elderly individuals: results from the ilSIRENTE study	Clinical Nutrition (H-index 121)	Article	Landi F et al	2012	647
7	Clinical definition of sarcopenia	Clinical Cases in Mineral and Bone Metabolism	Review	Santilli V et al	2014	607
8	Relative muscle mass is inversely associated with insulin resistance and prediabetes. Fundings from the Third National Health and Nutrition Examination Survey	Journal of Clinical Endocrinology & Metabolism (H-index 328)	Article	Srikanthan P et al	2011	568
9	The age-related loss of skeletal muscle mass and function: Measurement and physiology of muscle fiber atrophy and muscle fiber loss in humans	Aging Research Reviews (H-index 98)	Review	Wilkinson DJ et al	2018	479
10	Sarcopenia in older adults	Current Opinion in Rheumatology (H-index 102)	Review	Walston JD	2012	457

### 3.5 Keywords analysis

**Figure 6:**
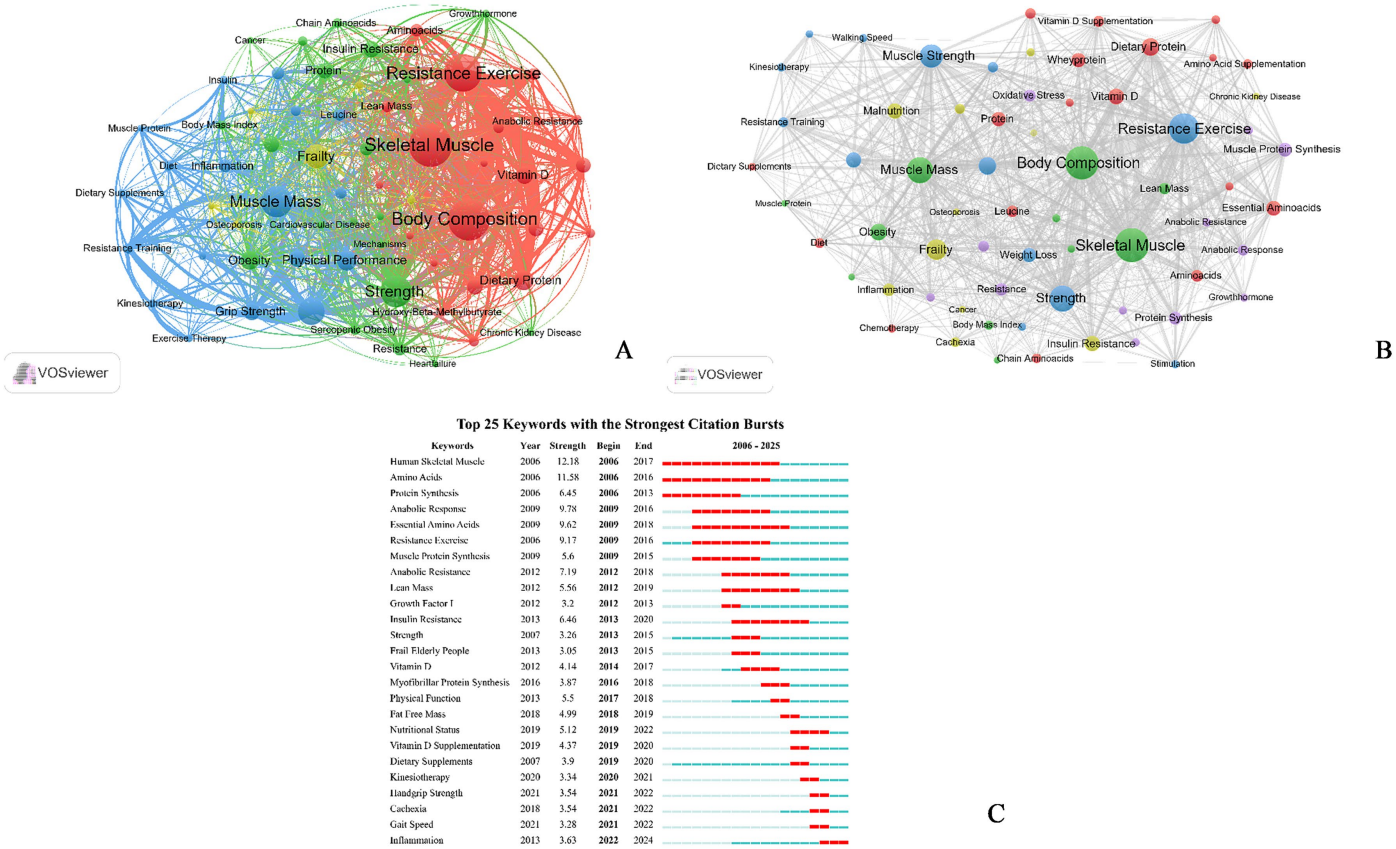
Visualization in research of exercise and nutrition for sarcopenia from 2005–2025 **(A)** Network visualization of the keywords co-occurrence analysis, the size of the nodes represents frequency; **(B)** the clustering map of keywords; **(C)** cluster analysis of top 10 keywords with strongest citation bursts from 2005 to 2025.

The keyword is the author’s refining and summarizing of the article’s content, which can reflect the core content of the article. By observing its historical development process and discovering unknown words, we can identify the changes and future trends of research hotspots in this field. High-frequency keywords can provide insight into the research areas that are currently receiving the most attentions, and frequent emerging keywords over time can offer indications of the cutting-edge topics that are being discussed in a given period. By visualizing and analyzing the keywords in all collected publications and constructing a network, 283 nodes and 475 links were obtained. In this analysis, 15 times or more are set as high-frequency keywords, and VOSviewer is used to present the co-occurrence network of high-frequency keywords. The thickness of the node connection is positively related to the co-occurrence frequency of the nodes at both ends of the link. A total of 83 keywords were selected with frequency≥15, and 36 keywords with BCD ≥ 0.05 (Table 2 and Figure 6A); among these, “muscle strength” and “resistance exercise” are the hotspots in “exercise”; and “vitamin D,” “dietary protein” and “amino acids” are hotspots in “nutrition”; “skeletal muscle,” “body composition” and “muscle strength” are hotspots in “sarcopenia.” This result suggests that the primary objective of exercise and nutrition interventions is to enhance the skeletal muscle strength and mass, thereby improving physical functioning in patients with sarcopenia. Concurrently, the keyword “obesity” suggests that the association between obesity and sarcopenia should also be emphasized.

**Table 2:** Top keywords with a frequency ≥ 15 in research for exercise and nutrition of sarcopenia from 2005 to 2025.

Keywords	Frequency	BCD	Node degree
Skeletal muscle	231	0.13	12
Body composition	197	0.15	9
Resistance exercise	149	0.11	7
Muscle strength	115	0.13	5
Vitamin D	65	0.14	5
Dietary protein	58	0.19	9
Protein intake	50	0.21	7
Amino acids	50	0.18	8
Obesity	44	0.16	8
Muscle protein synthesis	43	0.17	9

The 65 most significant keywords filtered for research purposes, were included in the cluster visualization analysis (Figure 6B). There are five clusters: cluster 1 (Protein), cluster 2 (Body Composition), cluster 3 (Resistance Exercise), cluster 4 (Frailty) and cluster 5 (Metabolism). Cluster 1 (red) is associated with diet and nutrition about sarcopenia, particularly “protein,” “vitamin D” and “creatine supplementation.” Cluster 2 (green) is primarily concerned with the evaluation of indices pertinent to sarcopenia, including “muscle strength” and “muscle mass.” Cluster 3 (blue) is primarily concerned with exercise for sarcopenia, mainly “resistance exercise.” Cluster 4 (yellow) is associated with diseases or manifestations related to sarcopenia, particularly “sarcopenic obesity,” “inflammation” and “osteoporosis.” Cluster 5 (purple) is some physiological processes which may related to sarcopenia, particularly “metabolic syndrome.”

In essence, keyword burst analysis analyzes how the frequency of keyword occurrences changes over a given period. The results are presented in Figure 6C. In the initial 5 years, the keywords of “human skeletal muscle,” “amino acids,” and “resistance exercise” burst out, which indicated more in the treatment of sarcopenia, and concentrated on the diagnosis of sarcopenia through the measurement of skeletal muscle. As the study advanced further, it began to explore more alternative treatment of sarcopenia, like the keywords of “vitamin D supplementation,” and the valuation of sarcopenia through the measurement of physical function. Meanwhile, sarcopenia has been associated with the keyword “lean mass,” “fat free mass” and “physical function.” In the final 5 years, the keywords have been more concentrated on “gait speed,” “cachexia” and “inflammation.” In those publications, researchers have focused on “cachexia” and “inflammation,” indicating that researchers are focusing on links between other diseases or pathologies and sarcopenia. Another research hotspot of sarcopenia is “gait speed,” which is one of the important metrics for assessing sarcopenia.

## 4 Discussion

Sarcopenia is a generalized skeletal muscle disease that is estimated to affect 10–16% of the elderly population worldwide (6, 27). With the increasing age globally, this percentage is expected to rise. The rehabilitation process of sarcopenia is closely associated with implementation of exercise and nutrition (28–31). As the number of studies in this field continues growing, it is important for researchers to have a current understanding of the developments and hotspots. Bibliometrics, as a rapidly growing and emerging discipline, allows for the quantitative analysis of huge amount of literature, and provide with more precise information about the evolution of research directions and frontiers (32–34). We performed a bibliometric analysis of the publications from WOSCC and Scopus database on exercise and nutrition for sarcopenia from 2005 to 2025 using CiteSpace and VOSviewer software, the current status, hotspots and frontiers trends in this field were then summarized.

A total of 886 publications including 601 articles and 285 reviews, related to exercise and nutrition for sarcopenia from 2005 to 2025 were retrieved by searching WOSCC and Scopus database. The number of annual publications on exercise and nutrition for sarcopenia showed an overall upward trend inspite of fluctuation slightly in some years. In 2005, there was no publication, but by 2023, the number of publications grew to 108, indicating a notable increase in researcher interest in this field. The number of publications has increased markedly since 2015 and will continue to increase in the future, indicating a growing interest in this field among researchers. With regards to national/regional publications, the USA, South Korea and Japan were the top three countries with highest number of publications, all of which have large elderly populations. As a result of increasing life expectancy and declining mortality, the number of aged persons (≥65) in China will be 217 million in 2024 (15.4% of the total population), and is expected to reach 402 million in 2040(>20% of the total population). The percentage of aged persons (≥65) in South Korea and Japan are about of 20 and 29.3% in 2024, separately (35).

It can be observed through the network that inter-country cooperation is most prevalent among developed countries, such as the USA, South Korea, Germany, Sweden, Spain and China, all of which have high centrality rankings. In recent years, China has demonstrated a notable surge in the number of publications, which may be attributed to the country’s transition into an aging society, and its growing emphasis on the elderly health. During the global pandemic of COVID-19, Asian countries, led by China and others, advocated for the advancement of healthcare services and the establishment of a resilient health-care system for the elderly. This was done in order to ensure the continued provision of quality healthcare in face of emerging and heightened demands during challenging times (36).

The findings indicated that research in exercise and nutritional interventions for sarcopenia may be distributed unevenly across geographical regions, characterized by a lack of collaboration and communication, and underdeveloped in most regions. A total of 1,341 institutions have published in this field over the past 20 years. The majority of the institutions that feature in the top 10 are located in developed countries in North America and Europe. Notably, the institution with the highest number of publications is from Italy, with the majority of North American and Australian institutions comprising the top 10 in the centrality rankings. This suggests that some institutions in European countries engage in less international communication and collaboration. In publication citations, institutions from Italy and the USA accounted for the majority, which suggests that these institutions may have a robust academic foundation in this field, making them a well-known source of expertise. In the last two decades, European countries have established EWGSOP2 (7) and Asian countries have established AWGS2 (4), facilitating communication and updating the consensus on the diagnosis and treatment of sarcopenia. However, global collaborative research on sarcopenia is still confronted with a number of challenges, including inconsistent definitions, geographic variations, and a lack of high-quality evidence, insufficient funding, and the absence of internationally recognized operational definitions. It is imperative that these obstacles be surmounted through international collaboration and multidisciplinary efforts.

Of the cited publications, the most frequently referenced publication was authored by EWGSOP and IWGS, which is an international authority in the field of clinical nutrition and metabolism, followed by the European Society for Clinical Nutrition and Metabolism (ESPEN), which has published several guidelines and consensus documents related to clinical nutrition, covering areas such as geriatric nutrition, oncology nutrition (37). “Prevalence of and interventions for sarcopenia in aging adults: a systematic review. Report of the International Sarcopenia Initiative (EWGSOP and IWGS)” is the most frequently cited review, and “Protein intake and exercise for optimal muscle function with aging: Recommendations from the ESPEN Expert Group” is the most frequently cited article. Through the publication of consensus documents and diagnostic criteria, EWGSOP, IWGS and ESPEN facilitate international research and collaboration on myasthenia gravis and promotes scientific advances in the field. Their work has had a profound impact on the clinical diagnosis and treatment of myasthenia gravis. Its definitions and diagnostic criteria are widely used in clinical practice and research (2, 3).

Keywords are indicative of the most prevalent and current areas of research interest. Among the high-frequency keywords searched skeletal muscle strength and mass are the key indicators that have an indispensable role in sarcopenia. The objective of exercise or nutritional intervention is to increase skeletal muscle mass and strength. Resistance exercise a training method enhancing muscular strength and endurance by overcoming external resistance is widely used in fitness and rehabilitation as well as to improve athletic performance. It is a hot research topic and the best-recommended exercise for sarcopenia (38–40). The potential use of vitamin D as a nutritional intervention for patients with oligomyelitis is currently under investigation. Nevertheless nutritional supplementation has been an efficacious intervention for sarcopenia with leucine supplementation in particular exerting a pronounced effect on muscle mass in older adults with sarcopenia (27, 41). Whey protein has been demonstrated to possess the capacity to stimulate muscle protein synthesis to a greater extent than other proteins including casein and soy. And it regulates muscle mass and body composition during the aging process. The combination of age-appropriate exercise and whey protein supplementation has the potential to not only enhance muscle mass and strength but also to improve other factors that contribute to the health of older adults with sarcopenia (42–44). Creatine monohydrate supplementation combined with resistance training provides some anti-sarcopenic benefits and has favorable effects on improving some aspects of cognitive function for older adults (45–49).

A substantial body of research has demonstrated that a combination of exercise and nutritional interventions has the potential to be highly efficacious in prevention and treatment of sarcopenia (31, 50–53). In cluster analysis of keywords, there had five clusters. Cluster 1 and cluster 3 includes interventions for sarcopenia, mainly resistance exercise and nutritional support. Cluster 2 mainly includes some criteria for the evaluation and diagnosis of sarcopenia. Cluster 4 deals with diseases and related manifestations associated with sarcopenia. Of these, more research has been done on obesity associated with sarcopenia. A new form of obesity among older adults has been identified, characterized by high fat mass and low muscle mass, which is known as sarcopenic obesity (a concurrent decrease in muscle mass and function, and an increase in body fat). The management of sarcopenic obesity is to integrate exercise and nutritional interventions with inducing a negative energy balance, thereby reducing body fat while maintaining or increasing muscle mass and function (54). There had other emerging trends in sarcopenia, such as osteosarcopenia (OS) and steatosarcopenia. OS, a dual condition of osteoporosis and sarcopenia, poses significant health risks. Patients with OS have a higher risk of malnutrition and adverse outcomes, including falls, fractures, and disability, compared to those with only sarcopenia or osteoporosis. This is because they suffer from both muscle and bone deterioration. The treatment involves a multifaceted approach, encompassing exercise (progressive resistance and balance training, 2–3 times per week), nutritional enhancement (adequate vitamin D, 1–1.5 g/kg/d protein, calcium, creatine), and drug therapy (anti-osteoporosis medications such as deslumab and zoledronic acid) (55). Steatosarcopenia, a condition characterized by the loss of mass or skeletal muscle strength and performance associated with the excessive deposition of ectopic reserve fat in muscle tissue, in the same individual, not necessarily related to excess fat total body mass (56).

Therefore, during nutritional intervention, it is essential to monitor and regulate both body weight and body fat. In addition, many chronic diseases related to sarcopenia also deserve attention. Cluster 5 contains key words about some potential mechanisms of sarcopenia. Keyword burst analysis showed the hot keywords in exercise and nutrition interventions for sarcopenia in the last 10 years were vitamin D supplementation, physical function, fat free mass, nutritional status, gait speed and inflammation. In the existing consensus documents on the diagnosis and treatment of sarcopenia, physical condition, lean weight, fat free weight and gait speed are important diagnostic and therapeutic criteria (1, 4). Gait speed is an important functional index in the diagnostic criteria of sarcopenia. According to the recommendations of the AWGS in 2019, gait speed less than 0.8 m/s is considered as one of the potential diagnostic criteria for sarcopenia (4). Through exercise intervention and nutrition management, the gait speed of patients with sarcopenia can be significantly improved, falls risk can be reduced and of life quality can be improved. For mechanisms, it has been hypothesized that oxidative stress plays a pivotal role in pathogenesis of skeletal muscle atrophy associated with sarcopenia (57, 58). There is a close association between anabolic resistance, insulin resistance and sarcopenia. Anabolic resistance and insulin resistance are important pathophysiological mechanisms of sarcopenia. They interact and jointly aggravate the progression of sarcopenia. Anabolic resistance may be one of the important pathophysiological mechanisms of sarcopenia, which mainly leads to the decline of muscle mass and strength (59, 60).

This study analyzed the global research trends for exercise and nutrition for sarcopenia, and the improving of three key elements (muscle mass, muscle power and muscle strength) will be the main intervention targets in the future study. However, sarcopenia is not only a stand-alone health problem, also an important complication that requires attention in management of multiple chronic diseases, such as chronic kidney disease (CKD), diabetes, inflammatory disease, malignant tumor, chronic obstructive pulmonary disease (COPD), inflammatory bowel disease (IBD), liver cirrhosis, Chronic wasting disease (tuberculosis). The presence of chronic diseases is often accompanied by an enhanced inflammatory response and protein catabolism. The effective management of chronic diseases has been demonstrated to reduce the body’s inflammatory response, thereby playing a significant role in preserving muscle mess and maintaining muscle strength and function (61). And global collaboration on research will be needed in enhancing the efficacy of future research, policy development in sarcopenia management, from phenotype to pathogenesis, from treatment to prevention.

There were some limitations in this study. Our search was confined to the WOSCC and SCOPUS database with three searching terms, and we exclusively considered English literature published from the last decade, and publications in other languages were not included. Furthermore, the quality of the publications was not taken into account, and all contributions were given equal weight. It is imperative to recognize that while the WoS Core Collection and SCOPUS database are an authoritative and comprehensive database, and tools like VOSviewer and CiteSpace are widely used software, their utilization introduces potential biases and limitations.

## 5 Conclusion

This study provides a thorough analysis of global research trends in sarcopenia over the past decade. A notable increase was observed in the adoption of integrated approaches that combine nutritional interventions with exercise-based therapies for treating sarcopenia. The focus of research in this field has shifted from addressing symptoms to exploring the underlying mechanisms. Furthermore, exercise and nutritional interventions are increasingly being utilized to address the disease burden and manage multiple chronic conditions associated with sarcopenia. Driven by the global aging population, this research has underscored the need for enhanced international cooperation.

## Data Availability

The original contributions presented in the study are included in the article/Supplementary material, further inquiries can be directed to the corresponding author.
