# Supplementary material for: Global research trends in sarcopenia: a bibliometric analysis of exercise and nutrition (2005–2025)
Source: Front Nutr. 2025 May 16;12:1579572. doi: 10.3389/fnut.2025.1579572 (PMC12122333; doi:10.3389/fnut.2025.1579572)
Supplement: Supplementary file 1 [file Data_Sheet_1.docx]

**Supplementary material:**

**Table S1.** Ranking of country/regional in publications amount and BCD in exercise and nutrition of sarcopenia from 2005-2025.

**Table S2** Ranking of research institutions in publications amount and BCD in exercise and nutrition of sarcopenia from 2005-2025.

**Table S3** Journals with more than 10 publications of research in exercise and nutrition of sarcopenia from 2005-2025.

**Table S4** The top ten journals in citations of research in exercise and nutrition of sarcopenia from 2005-2025.

**Table S1.** Ranking of country/regional in publications amount and BCD in exercise and nutrition of sarcopenia from 2005-2025.

| **Ranking of Publications** | | | | | **Ranking of BCD** | | | | |
| --- | --- | --- | --- | --- | --- | --- | --- | --- | --- |
| Rankings | Country/Regional | Publications | BCD | Node Degree | Rankings | Country/Regional | Publications | BCD | Node Degree |
| 1 | the USA | 148 | 0.08 | 3 | 1 | Germany | 26 | 0.73 | 14 |
| 2 | South Korea | 101 | 0.06 | 2 | 2 | Poland | 3 | 0.42 | 9 |
| 3 | Japan | 99 | 0.01 | 8 | 3 | Scotland | 8 | 0.38 | 6 |
| 4 | Italy | 96 | 0.06 | 3 | 4 | Croatia | 3 | 0.37 | 7 |
| 5 | the UK | 78 | 0 | 1 | 5 | Sweden | 24 | 0.35 | 7 |
| 6 | China | 77 | 0.09 | 4 | 6 | Switzerland | 19 | 0.3 | 10 |
| 7 | Canada | 65 | 0.07 | 8 | 7 | Mexico | 8 | 0.3 | 10 |
| 8 | Australia | 64 | 0.05 | 3 | 8 | Denmark | 5 | 0.29 | 5 |
| 9 | the Netherlands | 53 | 0.01 | 7 | 9 | Saudi Arabia | 4 | 0.28 | 5 |
| 10 | Spain | 50 | 0.2 | 3 | 10 | Spain | 50 | 0.2 | 3 |

**Table S2** Ranking of research institutions in publications amount and BCD in exercise and nutrition of sarcopenia from 2005-2025.

| **Ranking of Publications** | | | | | **Ranking of BCD** | | | | |
| --- | --- | --- | --- | --- | --- | --- | --- | --- | --- |
| **Institution** | **Amount** | **BCD** | **Node Degree** | **Rankings** | **Institution** | **Amount** | **BCD** | **Node Degree** | **Rankings** |
| University of Texas System（USA） | 17 | 0.05 | 7 | 1 | University of Alberta(CAN) | 12 | 0.11 | 11 | 1 |
| Catholic University of the Sacred Heart (IT) | 16 | 0.02 | 12 | 2 | McGill University(CAN) | 6 | 0.09 | 6 | 2 |
| McMaster University（CAN） | 16 | 0.03 | 2 | 3 | University of Amsterdam(NL) | 6 | 0.07 | 7 | 3 |
| IRCCS Policlinico Gemelli(IT) | 15 | 0.02 | 12 | 4 | Uppsala University(SE) | 10 | 0.06 | 16 | 4 |
| University of Melbourne (AUS) | 14 | 0.04 | 6 | 5 | Wageningen University & Research(NL) | 7 | 0.06 | 6 | 5 |
| University of Birmingham(UK) | 13 | 0 | 1 | 6 | Institut National de la Sante et de la Recherche Medicale (FRA) | 3 | 0.06 | 10 | 6 |
| National Research Institute for Agriculture, Food and Environment(FRA) | 12 | 0 | 10 | 7 | Alberta Health Services (CAN) | 2 | 0.06 | 2 | 7 |
| University of Alberta(CAN) | 12 | 0.02 | 11 | 8 | University of Texas System（USA） | 17 | 0.05 | 7 | 8 |
| Vrije Universiteit Amsterdam(NLD) | 12 | 0.11 | 3 | 9 | Karolinska Institutet (SE) | 11 | 0.05 | 7 | 9 |
| University of Texas Medical Branch Galveston(USA) Karolinska Institutet (SE) | 12 | 0.03 | 5 | 10 | CIBER - Centro de Investigacion Biomedica en Red (ES) | 11 | 0.05 | 17 | 10 |

**Table S3** Journals with more than 10 publications of research in exercise and nutrition of sarcopenia from 2005-2025

| Journals | Publications | IF_2024_ | Quartile in category |
| --- | --- | --- | --- |
| Nutrients | 76 | 4.8 | Q1 |
| Clinical Nutrition | 44 | 6.6 | Q1 |
| BMC Geriatrics | 25 | 3.4 | Q2 |
| Journal of Cachexia Sarcopenia and Muscle | 19 | 9.4 | Q1 |
| Journal of Nutrition Health & Aging | 18 | 4.3 | Q1 |
| Frontiers in Nutrition | 14 | 4 | Q2 |
| Current Opinion in Clinical Nutrition and Metabolic Care | 13 | 3 | Q1 |
| Clinical Interventions in Aging | 12 | 3.5 | Q2 |
| Experimental Gerontology | 12 | 3.3 | Q2 |
| Journal of the American Medical Directors Association | 12 | 4.2 | Q1 |
| Journal of Clinical Medicine | 11 | 3 | Q1 |
| Proceedings of the Nutrition Society | 11 | 7.7 | Q1 |
| BMJ Open | 10 | 2.4 | Q1 |
| Maturitas | 10 | 3.9 | Q1 |
| PLoS One | 10 | 2.9 | Q1 |

**Table S4** The top ten journals in citations of research in exercise and nutrition of sarcopenia from 2005-2025

| **Journals** | **Citations** | **IF_2024_** | **Quartile in category** |
| --- | --- | --- | --- |
| American Journal of Clinical Nutrition | 534 | 6.5 | Q1 |
| Age and Ageing | 524 | 6 | Q1 |
| Journals of Gerontology - Series A Biological Sciences and Medical Sciences | 512 | 4.3 | Q1 |
| Journal of the American Medical Directors Association | 505 | 4.2 | Q2 |
| Clinical Nutrition | 491 | 6.6 | Q1 |
| Journal of the American Geriatrics Society | 422 | 4.3 | Q1 |
| Journal of Nutrition, Health and Aging | 411 | 4.3 | Q1 |
| PLoS ONE | 387 | 2.9 | Q1 |
| Journal of Cachexia, Sarcopenia and Muscle | 381 | 9.4 | Q1 |
| Nutrients | 361 | 4.8 | Q1 |
